# Blockade of high-mobility group box 1 attenuates intestinal mucosal barrier dysfunction in experimental acute pancreatitis

**DOI:** 10.1038/s41598-017-07094-y

**Published:** 2017-07-28

**Authors:** Xia Chen, Hong-Xian Zhao, Chao Bai, Xiang-Yu Zhou

**Affiliations:** 1 Department of Gastroenterology, The Affiliated Hospital of Southwest Medical University, Luzhou City, P.R. China 646000; 2 Department of Histology and Embryology, Southwest Medical University, Luzhou City, P.R. China 646000; 3 Department of Vascular and Thyroid surgery, The Affiliated Hospital of Southwest Medical University, Luzhou City, P. R. China 646000

## Abstract

The release of inflammatory cytokines, that plays a dominant role in local pancreatic inflammation and systemic complications in severe acute pancreatitis (SAP). High-mobility group box 1 (HMGB1) is implicated in the mechanism of organ dysfunction and bacterial translocation in SAP. This current study aims to investigate possible role of HMGB1 in the intestinal mucosal barrier dysfunction of SAP, and the effect of anti-HMGB1 antibody treatment in intestinal mucosal injury in SAP. Our data revealed that the HMGB1 expression was significantly increased in AP mice induced by caerulein and LPS, and the inhibition of HMGB1 played a protective role in intestinal mucosal barrier dysfunction, reduced the serum level of other proinflammatory cytokines include IL-1β, IL-6, TNF-α. Next we investigated the downstream receptors involving in HMGB1 signaling. We found that the expressions of toll-like receptor (TLR) 4 and TLR9 were elevated in ileum of AP mice, the administration of HMGB1 neutralizing antibody significantly reduced the TLR4 and TLR9 expression. It was concluded that HMGB1 contributed the mechanism to the intestinal mucosal barrier dysfunction during AP. Blockade of HMGB1 by administration of HMGB1 neutralizing antibody may be a beneficial therapeutic strategy in improving intestinal mucosal barrier dysfunction in SAP.

## Introduction

Severe acute pancreatitis (SAP) is not only a local inflammatory disease of the pancreas, but also a systemic disease involving multiple organs. It is noted that the integrity of intestinal barrier is closely related to the degree of severity in acute pancreatitis (AP) and intestine is not merely a target organ of systemic inflammatory response syndrome (SIRS) but the origin of systemic inflammation^1^. The mechanisms of the intestinal barrier dysfunction in SAP are not definitely clear yet. Available evidences demonstrated intestinal barrier dysfunction in SAP might be associated with the release of inflammatory cytokines, ischemia-reperfusion injury, intestinal immunologic disorder, gut hypo-motility, long-term fasting, and apoptosis.

The important role of inflammatory mediators in SAP with SIRS was obtained increasing attention since 1988. Pro-inflammatory cytokines have been considering as the major risk factors in the development of SAP once they gain access to the systemic blood circulation^2^. At the early phase of SAP, the excessive leukocyte stimulation in pancreas induced the release of inflammatory cytokines includes tumor necrosis factor-alpha (TNF-α), interleukin-6 (IL6) *et al*.^3^. However, activation of polymorphonuclear granulocytes (PMNs) and of monocytes/macrophages is an early event during SAP. Cytokines such as TNF-α is rapidly cleared from the bloodstream, and sensitivity of its measurement seem strictly time dependent only in the onset of the disease^4^. Translating these pathogenic insights into clinical therapy has proved difficult, in part because of their narrow therapeutic window.

An alternative strategy would be to identify “late” mediators that may be clinically more accessible. High mobility group box 1 (HMGB1) protein, as a late mediator of endotoxin lethality, was found to be delayed released by cultured macrophages more than 8 hours after stimulation with endotoxin, TNF, or IL-1^5^. The importance of HMGB1 as a pro-inflammatory cytokine has been demonstrated in many inflammation-associated diseases^6^. Recent studies have shown HMGB1 may act as a key inflammatory mediator which participated in the development of SIRS and multiple organ damage in SAP^7, 8^. HMGB1 could influence the biological function of intestinal mucosa and seem to play a role in the development of intestinal barrier injury of SAP^9^. Anti-HMGB1 neutralizing antibody significantly improved the elevation of the serum amylase level and the histological alterations of pancreas and lung in SAP. Blockade of HMGB1 also significantly ameliorated the elevations of serum alanine aminotransferase and creatinine in SAP^10^. However, whether the blockade of HMGB1 could improve the intestinal barrier injury in SAP is not reported before.

Theoretically, there is a therapeutic window between symptom onset and the development of distant organ injury, when anti-inflammatory therapy may be of use. Elucidation of the key mediators in AP coupled with the discovery of specific inhibitors may make it possible to develop clinically effective anti-inflammatory therapy^11^. Thus, the purpose of this study was to determine the possible role of HMGB1 in the intestinal mucosal barrier dysfunction of AP. Moreover, whether the blockade of HMGB1 with a polyclonal antibody could ameliorate intestinal mucosal barrier dysfunction and its possible mechanism were also explored. These data would provide more information to understand the possible therapeutic potential of HMGB1 targeted intervention in intestinal barrier dysfunction in SAP.

## Results

### Elevated HMGB1 expression in the ileum of acute necrotizing pancreatitis (ANP) mouse model

**Figure 1:**
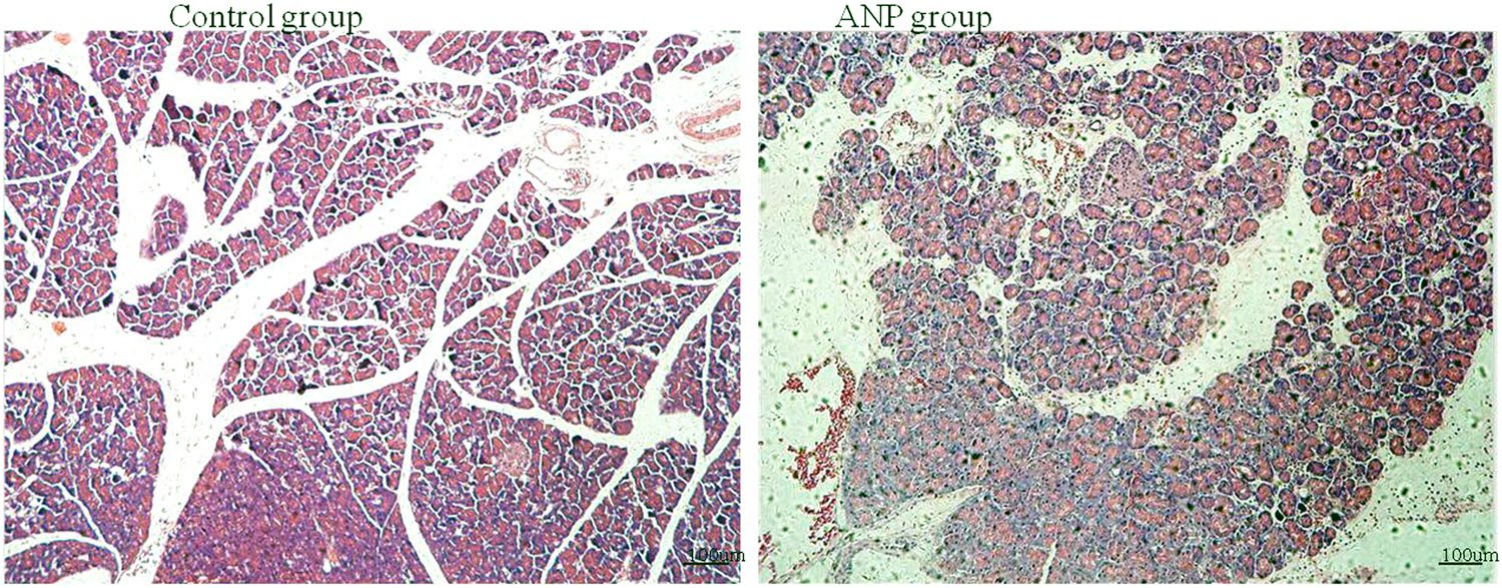
Representative H&E-stained sections of the pancreas. Pancreas sections from the control mice and the mice with ANP. The original magnification is x200.

**Figure 2:**
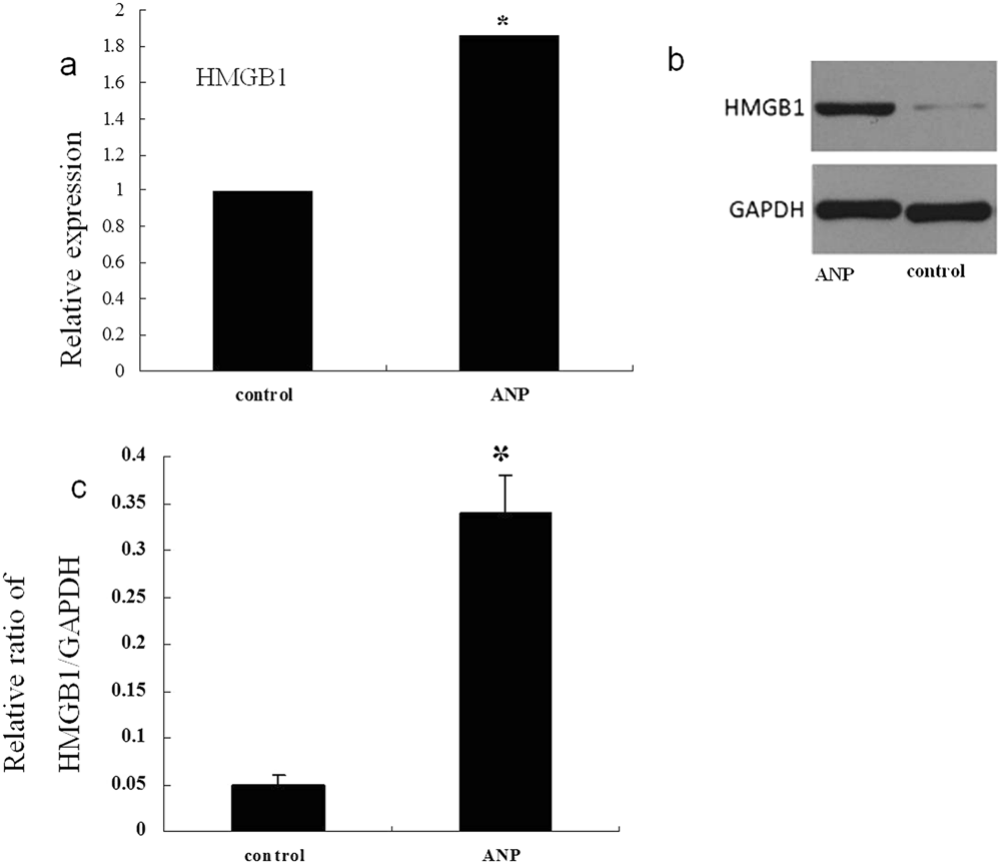
Elevated HMGB1 expression in the ileum of ANP. (**a**) Detection of HMGB1 mRNA expression by real-time PCR. Actin was used as a house-keeping gene. Relative expression differences of mRNA were normalized to endogenous actin expression and calculated using the 2^−∆∆CT^ method. The data of relative quantification from three independent experiments were expressed as means ± SD. *P < 0.05 denotes a significant difference compared with the control group. (**b**) HMGB1 protein expression by western blotting analysis in ileal tissues from control and ANP groups. GAPDH served as a loading control. (**c**) The relative ratios (Mean ± SD) of HMGB1/GAPDH are calculated based on the densities of bands on Western blots from three independent experiments. *P < 0.05 denotes a significant difference compared with the control group.

Severity of pancreatitis, HMGB1 expression was examined 12 h after induction of ANP. Induction of ANP was documented by histological findings of the pancreas (Fig. 1). Mice treated with caerulein and LPS all developed successfully to ANP. It was shown that the glands were grossly enlarged with visible areas of fat necrosis. Histopathological sections stained with H&E showed severe interstitial edema, pancreatic hyperemia, a mass of necrotic acinar cells and cell membrane dissolved. Histopathological sections stained with H&E showed a normal histological appearance of pancreas in the control mice. The expression of HMGB1 mRNA and protein in ileum were detected both at the mRNA level and protein level. Compared with the animals treated with PBS injection, real-Time Reverse-Transcriptase (RT) PCR data showed increased level of HMGB1 mRNA in ANP mice (Fig. 2a). Western blot analyses revealed the HMGB1 protein expression was also elevated in the ANP group compared with that in the control group (Fig. 2b,c).

### Anti-HMGB1 antibody treatment reduces serum IL-1β, IL-6, TNF-α level

**Figure 3:**
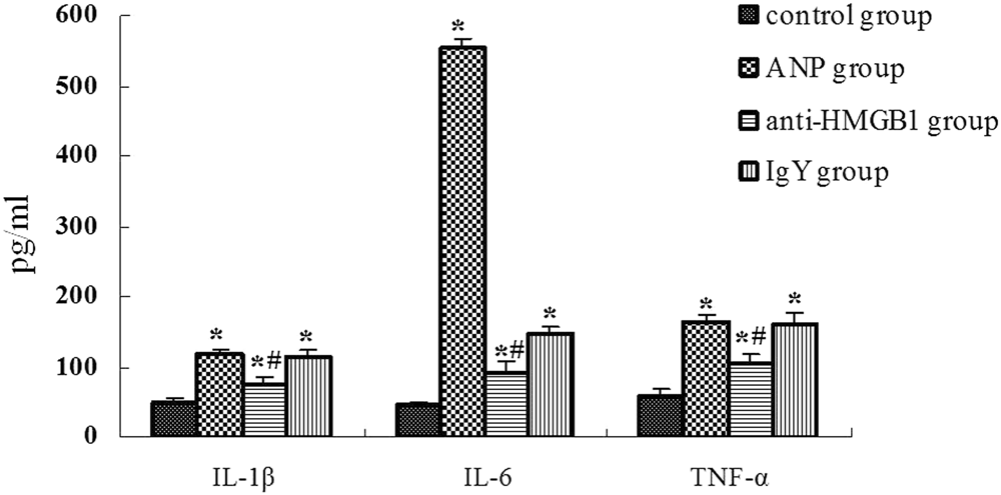
Serum IL-1β, IL-6, TNF-α levels detected by ELISA. Data expressed as the means ± SD. *P < 0.05 versus control group. ^#^ *P* < 0.05 versus ANP group.

Previous studies have reported that raised plasma levels of TNF-a, IL-6 and IL-1was present in patients with SAP^12^. These inflammatory cytokines have been demonstrated in association with the development of systemic complications of AP in early phase. In the present study, we investigated that the serum levels of IL-1β, IL-6, TNF-α were increased significantly in ANP group than that in control group (Fig. 3), this is consistent with previous reports. Besides, the possible effect of HMGB1 neutralizing antibody on these cytokines was investigated. As shown in Fig. 3, the serum levels of IL-1β, IL-6, TNF-α were decreased in the mice treated with HMGB1 neutralizing antibody compared with that in ANP group.

### Anti-HMGB1 antibody treatment improves the intestinal mucosal injury in ANP mouse

**Figure 4:**
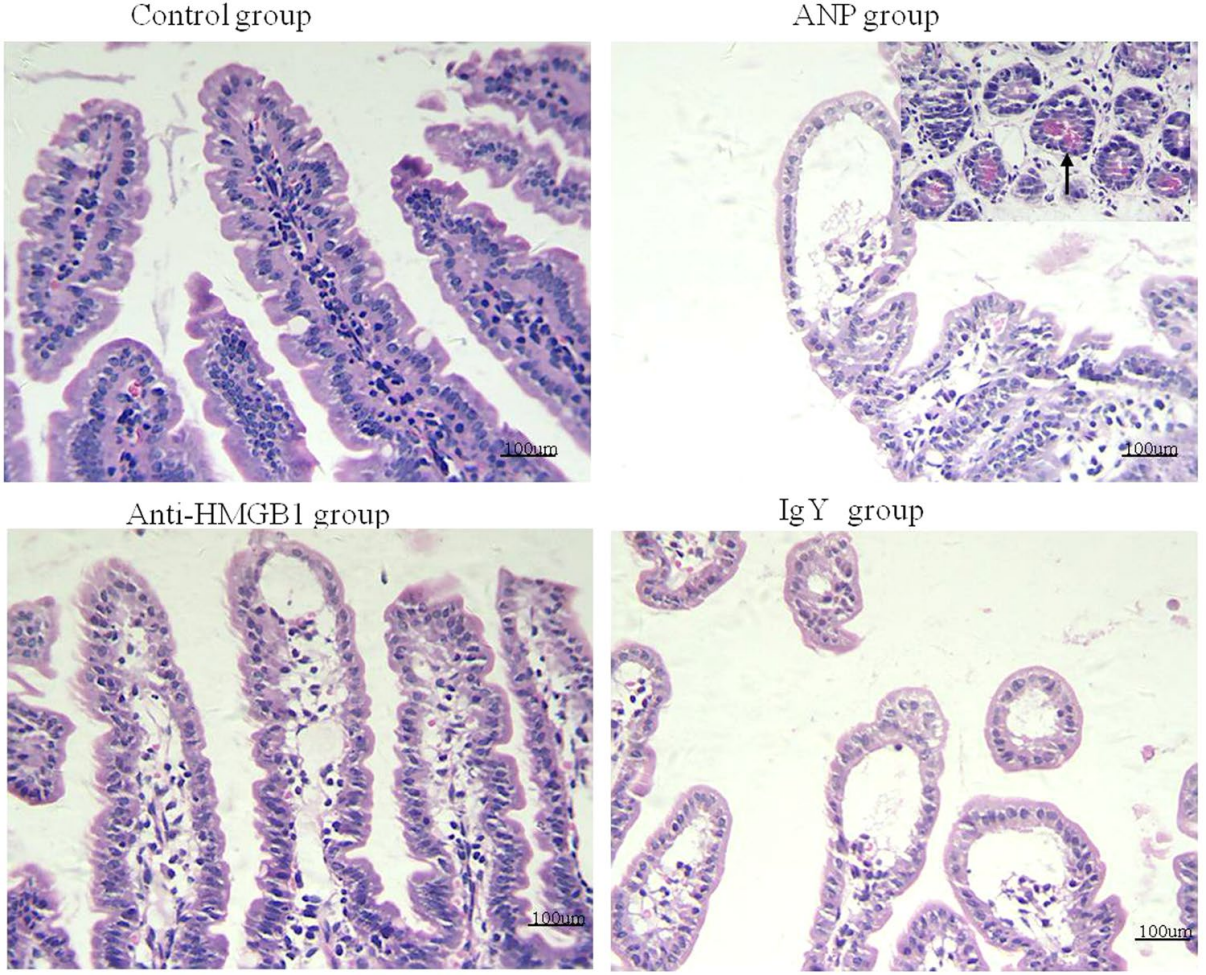
Representative H&E-stained sections of ileum. The original magnification is x400. Ileal mucosal injury was presented in ANP group characterized by irregularity and shedding of lamina propria with short, scanty, and denuded villi, increased paneth cells with more granules. Anti-HMGB1 antibody treatment attenuated AP-induced mucosal injury. (Black arrow: paneth cell with increased granules).

Sections of ileum were stained with HE and investigated under light microscope (Fig. 4). In control group, it was investigated that ileal epithelial cells arranged closely and regularly, villi were normal in shape, length, and number. Intestinal mucosal injury was presented obviously in ANP group. It is noted by irregularity and shedding of lamina propria with short, scanty, and denuded villi. Furthermore, increased paneth cells with more granules could be observed. Compared with animal in ANP group, histological investigation showed a relative recovery of mucosal injury of ileum in anti-HMGB1 group characterized by reduced irregularity and shedding of lamina propria and relative normal villi in shape, length, and integrity. Meanwhile, the mucosal injury in IgY group is similar with ANP group.

**Figure 5:**
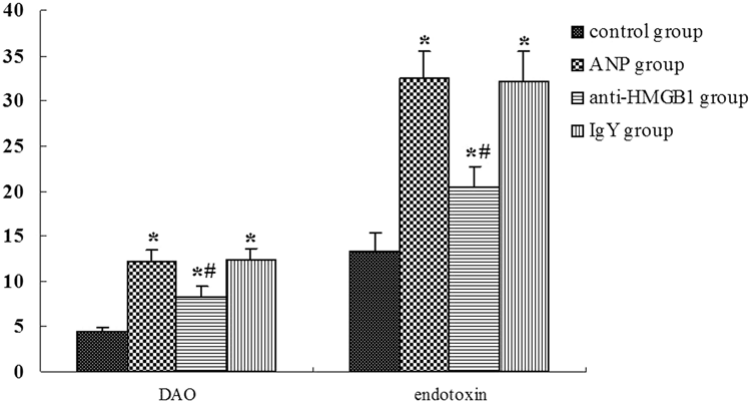
Serum DAO, endotoxin levels detected by ELISA. Data expressed as the means ± SD. *P < 0.05 versus control group. ^#^ *P* < 0.05 versus ANP group.

Diamine oxidase (DAO) is an intestinal mucosal enzyme that is in high activity in the mature upper villus cells of intestinal mucosa. The damage or necrosis of intestinal mucosa could induce DAO released into blood. Besides, failure of the gut barrier allows the passage of endotoxin from the gut lumen to systemic circulation. DAO and endotoxin core antibody were used as markers for the assessment of intestinal barrier function in our study. Results showed that mice with ANP had increased levels of serum DAO and endotoxin when compared with control mice (Fig. 5), confirmed that impaired intestinal mucosal barrier was present in AP mice. After the administration of HMGB1 neutralizing antibody, as shown in Fig. 5, the serum levels of DAO and endotoxin were significantly decreased.

**Figure 6:**
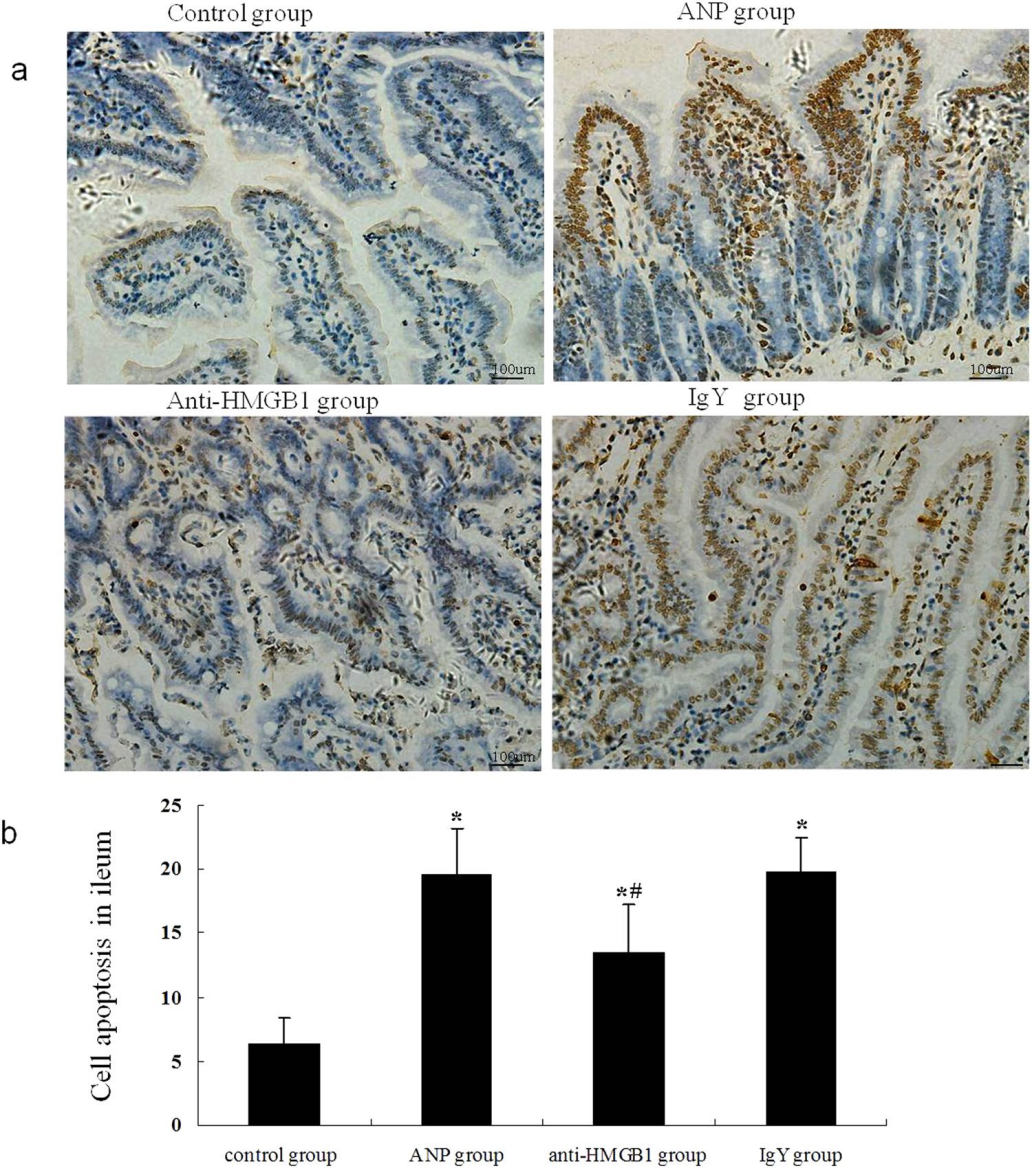
Apoptosis of ileal mucosal cells detected by TdT-mediated dUTP nick end labeling assay. (**a**) DAB staining, light microscopy, original magnification x200. The apoptotic cell was characterized by brown colored nuclei. (**b**) Comparison of ileal epithelial cell apoptosis in the different groups. The detection of apoptosis was semiquantified as defined in the “Materials and Methods” section. Data (IOD value) expressed as mean ± SD (n = 10 animals per group). *P < 0.05 versus control group. ^#^ *P* < 0.05 versus ANP group.

Intestinal epithelial apoptosis also contributes to mucosal barrier dysfunction in previous reports^13^. In present study, we investigated the apoptosis of intestinal mucosal cell by TdT-mediated dUTP nick end labeling (TUNEL) method. As exhibited in the Fig. 6a, we observed significantly increased labeling mucosal cells in mice with ANP. HMGB1 neutralizing antibody treatment attenuated the cell apoptosis in mucosa of ileum (Fig. 6a). Compared with animals in the control group, the integrated optical density (IOD) value of TUNEL staining of mucosal cell increased in ANP group (Fig. 6b). This difference was statistically significant. The IOD value of apoptosis in anti-HMGB1 group was reduced compared with that in ANP group accordingly (Fig. 6b).

### Anti-HMGB1 antibody suppresses the elevated expression of TLR4, TLR9

**Figure 7:**
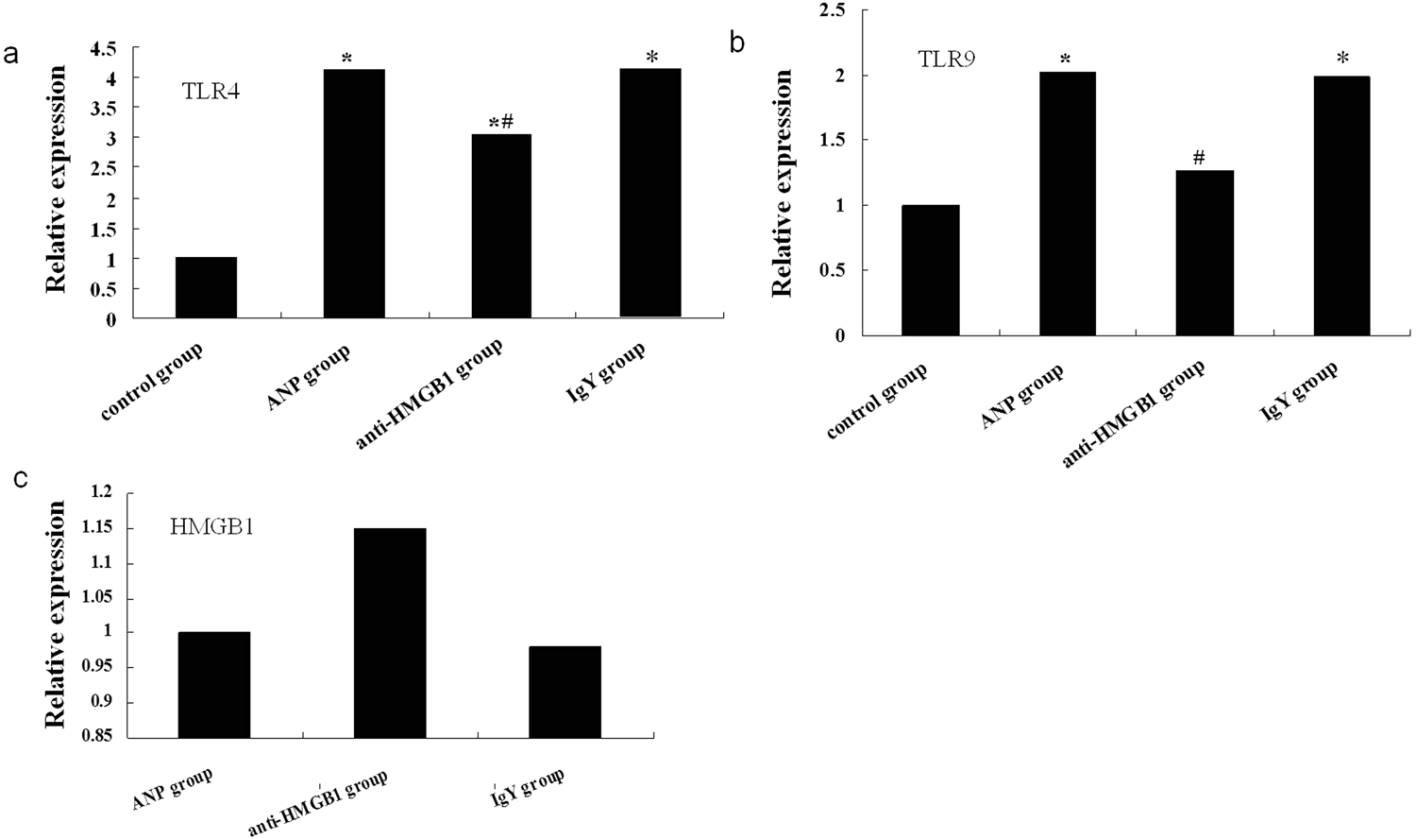
Anti-HMGB1 antibody treatment suppresses the elevated expressions of TLR4, TLR9 mRNA. (**a**) Detection of TLR4 mRNA expression by real-time PCR. Actin was used as a house-keeping gene. Relative expression differences of mRNA were normalized to endogenous actin expression and calculated using the 2^−∆∆CT^ method. (**b**) Detection of TLR9 mRNA expression by real-time PCR. (**c**) Detection of HMGB1 mRNA expression by real-time PCR. *P < 0.05 versus control group. ^#^ *P* < 0.05 versus ANP group.

**Figure 8:**
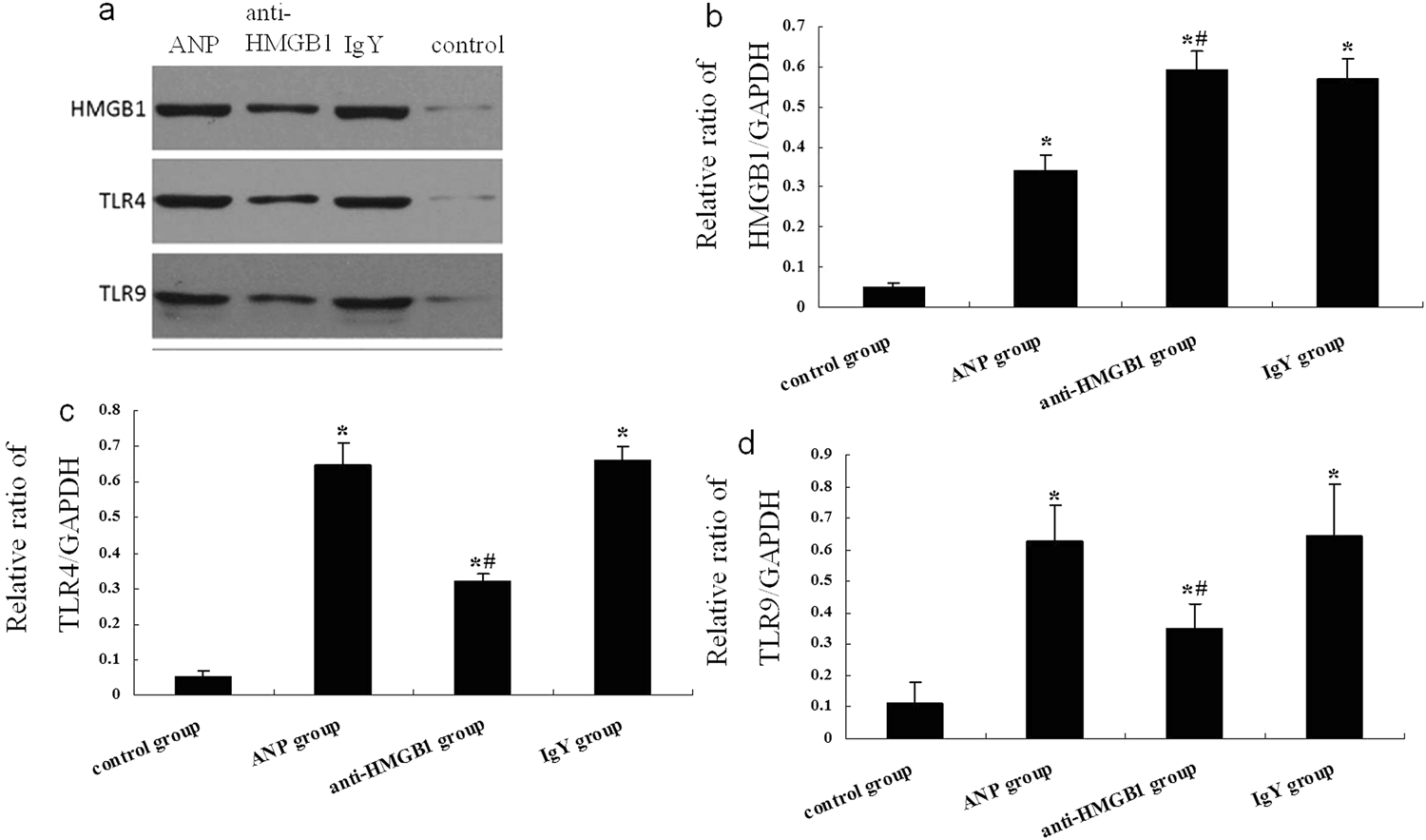
Anti-HMGB1 antibody treatment suppresses the elevated expressions of TLR4, TLR9 protiens. (**a**) Western blot showed the expression of HMGB1, TLR4, TLR9 proteins in ileal mucosa. GAPDH served as a loading control. (**b**) The relative ratio (Mean ± SD) of HMGB1/GAPDH. (**c**) The relative ratio (Mean ± SD) of TLR4/GAPDH. (**d**) The relative ratio (Mean ± SD) of TLR9/GAPDH. *P < 0.05 versus control group. ^#^ *P* < 0.05 versus ANP group.

Next, we further our investigation into the downstream receptors include TLR4, TLR9 involving HMGB1 signaling pathway, which are the main receptors of HMGB1 and expressed in intestinal epithelial cell to different degrees. As predicted, the expressions of TLR4 mRNA and proteins were elevated in ANP group compared to that in control group (Figs 7a and 8a,c). TLR4 has been demonstrated to participate in the organ dysfunction and bacterial translocation in SAP, and which may trigger the inflammatory response and function defensively against infection^14^. Administration of HMGB1 neutralizing antibody decreased the expression of TLR4 both at mRNA and proteins levels compared with ANP group, while unchanged in IgY treated AP mice (Figs 7a and 8a,c). Previous study demonstrated that TLR9 was expressed in resident immune cells of the pancreas and TLR9 antagonist could reduce pancreatic edema, inflammatory infiltrate, and apoptosis in AP^15^. But the role of TLR9 in intestinal mucosa barrier dysfunction of AP was not reported before. In this study, The TLR9 mRNA and protein expressions were increased in ANP group compared to that in control group (Figs 7b and 8a,d). Treatment with HMGB1 neutralizing reduced TLR9 mRNA and protein expressions compared with ANP group (Figs 7b and 8a,d). However, compared to mice with ANP, the HMGB1 mRNA level was changed little in anti-HMGB1 antibody-treated animals (Fig. 7c). But western blotting showed the expression of HMGB1 protein was decreased significantly in mice treated with HMGB1 neutralizing antibody (Fig. 8a,b).

## Discussion

Intestine is not only in charge of the digestion and absorption of nutrient, mostly, possesses the function of barrier to defy the harmful substances. The translocation of gut bacteria and endotoxin following intestinal mucosal barrier injury is a key event contributing to the severity of AP, even more, initiate and aggravate SIRS or multiple organ dysfunction syndrome (MODS), which are important factors influencing severity and mortality of AP^16^. The improvement of intestinal barrier injury therefore is of great significance in preventing the development of AP.

The increased DAO activity and endotoxemia are thought to be useful marker of intestinal mucosal barrier injury. The normal plasma DAO is primarily influenced by intestine and its basal plasma levels are positively correlated with the maturity and integrity of the intestinal mucosa^17^. Intestinal mucosal cells injury or necrosis would induce the release of DAO into peripheral blood. Endotoxin core antibody is normally present in very small amounts in the circulation. The increase of intestinal mucosal permeability in AP is accepted as the main cause of endotoxemia. In present study, serum levels of DAO and endotoxin core body were increased in mice with ANP, together with the histopathologic changes of ileal mucosal impairment, indicating the presence of intestinal mucosal barrier dysfunction during the development of AP. In addition, we found the cell apoptosis was increased in intestinal mucosal cells in ANP mice. Increased apoptosis in the intestinal epithelium was also thought to be associated with intestinal barrier dysfunction in experimental pancreatitis^18^. However, mechanism underlying intestinal mucosal barrier injury is not complete clarified, the detailed mechanism needs to be further investigated.

The key pathogenesis of intestinal mucosal barrier dysfunction in early phase of SAP is the excessive release of inflammatory cytokines and excessive systemic inflammatory response. The intestinal epithelium is an important source of pro-inflammatory mediators and play an important role in initiate the activation of neutrophils and release of inflammatory cytokines. Among these cytokines, HMGB1 is one of the important molecules of damage-associated molecular pattern molecules (DAMPs) that are the mediators of the systemic inflammatory response and cause further pancreatic damage^19^. Compared with early mediators such as TNF-α, IL-1, HMGB1 appears to mediate late lethality from endotoxic shock. HMGB1 is a potent mediator of inflammation secreted by activated monocytes/macrophages, and is passively released by necrotic or damaged cells^20^. Increasing evidences have demonstrated HMGB1 act as a critical molecular target in multiple human diseases including infectious diseases, acute lung injury, brain injury, liver disease, intestinal barrier disruption, vascular barrier disruption, precancerous lesions^21–23^. Previous studies showed HMGB1 was significantly higher in patients with SAP and experimental AP, participating in lung injury, liver injury, pancreatic tissue damage, intestinal barrier injury complicated by SAP, which seem to act as a key mediator for inflammation and organ failure in SAP^7^. In present study, the expression of HMGB1 mRNA and protein was both up-regulated in intestinal mucosa of AP mice complicated with intestinal barrier dysfunction. The result confirmed findings from earlier studies showing that HMGB1 contribute to the pathogenesis in intestinal barrier dysfunction in AP^9^.

HMGB1 may become the new target for therapeutic benefit while its biological function and the important role in multiple human diseases revealed by extensive researches. Inhibiting HMGB1, a late mediator of lethal systemic inflammation seems to represent a novel approach that may widen the therapeutic time window and lead to new strategies for the deleterious effects of the inflammatory process. In hemorrhagic shock and resuscitation (HS/R) animal model, the treatment of anti-HMGB1 neutralizing antibody could ameliorate HS/R-induced ileal mucosal hyperpermeability and bacterial translocation^24^. Anti-HMGB1 antibody also reversed LPS-induced gut barrier dysfunction in rats, demonstrating its protective effect on gut barrier dysfunction^21^. But in experimental SAP, the inhibition of HMGB1 with neutralizing antibody significantly improved the histological alterations of pancreas and lung injury, whereas worsened the bacterial translocation (especially gram-negative bacteria)^10^. Therefore, it is not clear whether the inhibition of HMGB1 protect the intestinal barrier dysfunction in SAP and further investigation was needed. On the basis of this information, we used HMGB1 neutralizing antibody to neutralize the extracellular HMGB1 in AP mice, explore the possible effects of neutralizing anti-HMGB1 antibody on intestinal mucosal barrier dysfunction in AP in present study. We found the alleviation of histopathological changes of ileal mucosal injury, decreased serum DAO and endotoxin levels after the administration of anti-HMGB1 antibody, suggesting the inhibition of extracellular HMGB1 could ameliorate the intestinal mucosal barrier dysfunction in AP. Besides, the cell apoptosis of intestinal epithelium was also decreased in anti-HMGB1 antibody treated mice. Apoptotic cells do not release HMGB1 even after undergoing secondary necrosis and partial autolysis^25^. The role of HMGB1 in apoptosis is not yet fully understood. Researches showed HMGB1 may have anti-apoptosis properties and participate in the regulation of apoptosis of mammary gland, retinoblastoma cells, submandibular glands while other studies reported HMGB1 induced apoptosis of melanocyte, cervical cancer cell *et al*.^26–29^. Another report found the apoptotic effect of HMGB1 depended on its concentrations: HMGB1 at low concentration promoted apoptosis, while reduced this apoptosis at high concentration^30^. In present report intestinal mucosal cell apoptosis was decreased in anti-HMGB1 antibody treated mice. But whether this anti-apoptotic effect is caused by the neutralizing role of anti -HMGB1 antibody to HMGB1 or the anti-inflammatory effect induced by HMGB1 blockade need more exploration.

Early proinflammatory cytokines such as TNF-α, IL-1β, and IL-6 could stimulated by immunological cells (macrophages, lymphocytes) in the course of AP^31^. HMGB1 is also positioned as a mediator of other inflammatory conditions associated with increased levels of TNF and IL-1, activate macrophages to release TNF and other proinflammatory cytokines. In the present study, significantly reduced serum TNF-α, IL-1β, and IL-6 levels were detected in the anti-HMGB1 antibody treated mice, indicating HMGB1 is an important regulator of these proinflammatory cytokines. The excessive release of inflammatory cytokines is thought to contribute to the induction of a systemic inflammatory response and multiple organ failure in patients with AP^32^. Hence, it is likely this anti-inflammatory effect of the blockade of HMGB1 relate to the regulation of other inflammatory cytokines, and moreover, relate to its protective role in intestinal barrier dysfunction in AP.

Studies have identified toll-like receptors (TLRs) such as TLR4, TLR9 have been shown to be involved in HMGB1 signaling^22^. TLRs are considered to be the key components of the innate immune system and have major roles in the initiation of the inflammatory response. The first mammalian family member of TLRs to be discovered was TLR4. HMGB1 could up-regulate TLR4 expression and activate the TLR4-mediated NF-κB signaling pathway to induce pancreatic injury while the pancreatic injury was significantly reduced in TLR4-deficient mice, showing that HMGB1-induced pancreatic injury is predominantly mediated by the TLR4 signaling pathway^33^. The activation of TLR4 signaling seems to be associated with SIRS, multiple organ dysfunction and intestinal bacterial translocation in AP^34^. TLR4 deficiency promoted repair of the liver and kidney injury, but aggravated the translocation of gram-negative bacteria to pancreas in mouse with SAP^14^. In this study we found the expression of TLR4 mRNA and protein was elevated in ileum of ANP mice, anti-HMGB1 antibody treatment reduced TLR4 expression, suggesting TLR4 may be the downstream signaling molecule of HMGB1 involved in the intestinal barrier dysfunction in AP, but the definite role of TLR4 in intestinal barrier dysfunction in AP is await to further research. Besides, the other member of TLR family, TLR9, its activity could be mediated by HMGB1 either, was also increased in intestine and could be reduced by anti-HMGB1 antibody intervention. TLR9 interact with cytosine-guanosine dinucleotide could affect the function of the intestinal immune system, which is essential for homeostasis of the intestinal immune system as it is required for the induction of counterregulating anti-inflammatory mechanisms^35^. TLR9 inhibition could decrease both pancreatic IL-1β expression and lung inflammation in experimental AP^15^. Taken together, our results suggest that HMGB1 may interact with TLR4 and TLR9 to mediate intestinal barrier dysfunction in AP, furthermore, the protective effect on intestinal barrier dysfunction exerted by the inhibition of HMGB1 may correlate with both TLR4 and TLR9. Gribar *et al*.^36^ reported that in experimental necrotizing enterocolitis, the intestinal expression of TLR4 and TLR9 are reciprocally related.TLR9 activation limited TLR4 signaling in enterocytes *in vitro* and within the intestinal mucosa *in vivo*. It seems that a kind of proper balance between TLR4 and TLR9 is required to maintain intestinal immune homeostasis. However, additional studies are needed to explore the relative roles of TLR4 and TLR9 signaling in intestinal mucosal dysfunction in AP.

In summary, we now report that the intestinal expression of HMGB1, TLR4 and TLR9 are elevated in intestinal mucosal in AP mice. The inhibition of HMGB1 by HMGB1 neutralizing antibody could ameliorate the intestinal mucosal barrier dysfunction, decrease serum level of other proinflammatory cytokines, reduce the expression of downstream receptors includes TLR4 and TLR9.These results demonstrated the potential of HMGB1 be a therapeutic target and the protection achieved from HMGB1 blockade for intestinal mucosal barrier dysfunction in SAP.

## Materials and Methods

### Animals

Male adult KM mice were weighing 20–25 g originally purchased, maintained, and bred in house at the Experimental Animal Center of Southwest Medical University (Luzhou, China). Ten male mice per group, were housed in rooms controlled temperature (21–24°C) and maintained light/dark cycle (12:12) for 1 week to acclimate the surroundings, with free access to tap water and standard laboratory chow. Before the induction of AP, mice were fasted for 12 h but had free access to water. The animal experiments were approved by The Animal Care and Welfare Committee of Southwest Medical University, and conducted according to the guidelines of the Local Animal Use and Care Committees of Luzhou as well as the National Animal Welfare Law of China.

### Establishment of ANP model and experimental design

Mice were randomly allocated into four groups as follow: ANP group (ANP animals only), control group, anti-HMGB1 group (ANP animals treated with HMGB1 neutralizing antibody) and IgY group (ANP animals treated with nonimmune chicken IgY). ANP mouse models were induced as previously described^37^. Briefly, ANP mouse was established with caerulein (sigma, St Louis, USA) at a dose of 50 µg/kg, by 13 consecutive hourly intraperitoneal (i.p.) injections, followed immediately by a single dose of 10 mg/kg LPS injection. PBS injection served as control. HMGB1 neutralizing antibody treated mice were injected intraperitoneally by anti-HMGB1 polyclonal antibody (Shino-Test, Tokyo, Japan) at a dose of 300 µg just after LPS injection. The neutralizing activity of anti-HMGB1 was confirmed in HMGB1-stimulated macrophage cultures by assay of TNF release. In the presence of anti-HMGB1 antibody, neutralizing antibody was defined as inhibiting 80% of HMGB1-induced TNF release. Nonimmune chicken IgY (Shino-Test, Tokyo, Japan) act as control antibody for HMGB1 neutralization was also at a dose of 300 µg injected intraperitoneally just after LPS injection. Studies were carried out at the same periods: twelve hours later.

### Histological examination

For assessing the changes occurring in the pancreatic tissue and ileal mucosa at the morphological level, pancreatic and ileal tissue specimens were fixed in 10% buffered formalin overnight and subsequently dehydrated through a graded ethanol series. Samples were paraffin-embedded. Tissue samples were cut into sections (5 μm). H&E staining was used for routine histological examination.

### Enzyme-linked immunosorbent assay (ELISA)

Twelve hours after LPS injection, blood was obtained by cardiac puncture, and the serum was collected and stored frozen at −80 °C. IL-1β, IL-6, TNF-α, DAO, Endotoxin core antibody were measured using commercially available enzyme-linked immunosorbent assay (ELISA)(Neobioscience, Beijing, China). All test steps were strictly according to the manufacturer’s instructions. Statistical analysis was by the Mann-Whitney U test.

### TdT-mediated dUTP nick end labeling (TUNEL)

Ileal mucosal apoptotic cells were detected by the TUNEL method (Roche Diagnostics, Bromma, Sweden). For the TUNEL reaction, tissue sections from ileum were dewaxed and rehydrated, then incubated for 30 minutes at 37 °C with proteinase working solution. The slides were placed in a plastic jar containing 200 mL of 0.1-mol/L citrate buffer (pH 6.0) and applied 350 W microwave irradiation for 5 minutes. The slides were rinsed twice with phosphate-buffered saline (PBS) and incubated with a mix solution composed of the enzyme terminal deoxynucleotidyl transferase and nucleotide mixture (label solution) in a humidified box in the dark for 60 minutes at 37 °C. For negative control, only 50 μL label solution was added. Then, the slides were incubated after adding 50 μL of converter peroxidase. Diaminobenzidine was used as the substrate for peroxidase, yielding the characteristic brown color for nuclei. For each test, negative controls were included. Semiquantitative results of apoptosis were calculated by integrated optical density (IOD) value measured by an Optimas image analysis system.

### Real-Time Reverse-Transcriptase (RT) PCR

Total RNA was extracted from stored ileum samples using an RNA simple total RNA kit (Tiangen biotech, Beijing, China) according to the manufacturer’s protocol. Genomic cDNA eraser and cDNA synthesis (Primescript RT reagentkit with cDNA eraser, Dalian, China) were also performed following the manufacturers’ protocols. The mRNA levels of HMGB1,TLR4,TLR9 were determined in a real-time quantitative RT-PCR using SYBR premix EX Taq (Takara Biotech, Dalian, China), iCycler thermal real-time PCR system (MJ research, MN,USA). Primers (Invitrogen, Carlsbad, CA, USA) for the amplification of the genes HMGB1, TLR4, TLR9 are presented in Table 1. Results were repeated in at least three independent RNA preparations. The expression levels were normalized to actin, which was used as an internal control gene and analyzed using the 2^−∆∆Ct^ method.

**Table 1:** Sequences of oligonucleotide primers used for RT-PCR.

Gene	Forward primer	Reverse primer
HMGB1	5′ - CGAGCATCCTGGCTTATC -3′	5′ - TCAGCTTGGCAGCTTTCT -3′
TLR4	5′ - TTTATTCAGAGCCGTTGG -3′	5′ - AGGCGATACAATTCCACC -3′
TLR9	5′ - CATCTTCTTCCGCTTGCT -3′	5′ - GCCCACTGATGCGATTGT -3′
Actin	5′ - AATCGTGCGTGACATCAAAGAG -3′	5′ - CCAAGAAGGAAGGCTGGAAAA -3′

### Western blotting analysis

The expression of ileal HMGB1, TLR4, TLR9 proteins were measured by western blot analysis. The protein was extracted by nuclear and cytoplasmic extraction reagents according to the instructions (Beyotime Institute of Biotechnology, Shanghai, China). Protein extracted from samples of 40 µg were run on 10% SDS-PAGE gels, and then transferred to polyvinylidene difluoride (PVDF) membranes (Beyotime Institute of Biotechnology, Shanghai, China).The membranes were incubated in TBST containing 5% non-fat dried milk (1 h, 25 °C),and incubated overnight at 4°C with primary antibodies to HMGB1, TLR4, TLR9 (1:1000,Cell Signaling Technology, Boston, USA). Subsequently, membranes incubated with HRP-conjugated anti-mouse secondary antibodies (1:3000, Beijing Zhongshan Biotechnology, Beijing, China) for 1 h at room temperature and visualized with an enhanced chemiluminescence assay detection kit (Beyotime Institute of Biotechnology, Shanghai, China). The bands were quantified using MultiGauge version 3.2 software. And the expression of the target protein was normalized to the level of GAPDH in the same sample.

### Statistical Analysis

Parametric values are expressed as the means ± SD if not otherwise indicated. Statistical analysis was performed by one-way analysis of variance (one-way ANOVA) and Student-Newman-Keuls test (q-test). *P *< 0.05 was considered statistically significant.
